# Estimation of ground reaction force direction in inline speed skating motion

**DOI:** 10.3389/fspor.2026.1782354

**Published:** 2026-04-13

**Authors:** Yuya Kimura, Toshiharu Yokozawa

**Affiliations:** Department of Sports Sciences, Japan Institute of Sports Sciences, Tokyo, Japan

**Keywords:** biomechanics, estimation method, force plate, ground reaction force, human motion tracking, motion capture system, speed skating, supporting leg posture

## Abstract

This study proposes and verifies a method to estimate the ground reaction force (GRF) direction from supporting-leg posture during speed skating. We hypothesized that GRF direction can be accurately estimated from supporting-leg posture. Two estimation methods were developed under the assumption that the reaction force acts perpendicular to the shoe sole. One method used the coordinates of the front and rear blade ends and the ankle joint center (Est1). In contrast, the other used the coordinates of the front and rear blade ends and the knee joint center (Est2). The magnitude of each GRF component was estimated by multiplying the resultant force measured by the force plates (FP) by the corresponding unit vector components calculated in Est1 and Est2. GRFs measured using FP were adopted as true values to verify the proposed methods. Sixteen experienced university speed skaters performed straight and curved inline speed skating trials, during which three-dimensional kinematic and FP data were recorded. The root mean square error (RMSE) between FP measurements and estimates from Est1 and Est2 was calculated for each force component. The largest observed error was approximately 10% of body weight. Across all skating motions and force components, Est2 consistently exhibited smaller RMSE values than Est1. Based on these results, Est2 was selected for further verification. For straight skating and the right leg during curve skating, bias values for all components were less than 8% of body weight. For the left leg during curve skating, larger biases were observed before and after the right blade contact; however, applying regression-based corrections substantially reduced these biases. Intraclass correlation coefficient analysis indicated agreement between true and estimated values for the X' and Z' components across all skating motions. For the Y' component, several time points showed no agreement with the true values. Nevertheless, because the Y' component magnitude was very small, its influence on calculations such as joint torque or joint power of the supporting leg was minimal. Overall, Est2 is suitable for these calculations.

## 1 Introduction

Speed skating is a competitive sport performed on ice. Measuring ice reaction forces acting on skaters clarifies force generation patterns and acceleration mechanisms during high-speed skating (1, 2). Such measurements also enable calculation of joint torque and power of the supporting leg, providing insights that may improve training and skating techniques (3, 4). However, force plates (FP) cannot be used on ice, which makes direct measurement of reaction forces difficult. Previous studies have addressed this issue by creating special instrumented blades worn by skaters (1, 2, 5–7). These blades incorporate welded components, including strain gauges, force sensors, cables, and data loggers. This equipment interferes with movement, making it difficult for skaters to perform optimally. Methods that allow skaters to use their standard shoes and blades while measuring force are advantageous for training and coaching. One promising approach involves the use of foot-pressure sensors. Using foot-pressure magnitude and center-of-pressure location data, the force magnitude and point of application can be estimated. Recently, wireless insole-type foot pressure sensors have been developed and applied to measure movements across various sports (8, 9). However, foot pressure data alone are insufficient to estimate the direction of the force application. Therefore, estimating force direction would enable force measurement without the use of special instrumented blades.

In gait research, numerous studies have estimated ground reaction force (GRF) direction using posture data obtained through motion capture systems (10–14). Similar approaches have been applied to activities of daily living, including stair climbing, obstacle negotiation (15), and stepping tasks (16). Additionally, Comparable methods have also been reported for active movements such as running, sidestepping, cutting, and jumping (17–21). Among these studies, Komaris et al. (18) estimated GRFs during running using supporting leg shank posture obtained via optical motion capture. Jiang et al. (22) estimated GRFs during walking using inertial measurement units (IMUs) attached to each segment of the supporting leg, including foot, lower leg, distal thigh, and proximal thigh. They reported a particularly high accuracy for methods using data from the lower legs and feet. Based on these studies, we hypothesized that GRF direction could be estimated from body segment posture data, particularly from the supporting leg, during speed skating. In speed skating, attempts have been reported during straight skating motions (23). However, there are only a few studies have estimated GRFs from posture data during speed skating. Furthermore, no reports concerning curve skating motions have been found. Therefore, it is necessary to determine whether methods previously used to estimate GRFs from supporting leg posture data are applicable to speed-skating motions.

Accordingly, this study aimed to propose and validate a method for estimating GRF direction from supporting leg posture during speed skating. We hypothesized that GRF direction can be accurately estimated from supporting leg posture. Furthermore, assuming that the ice reaction force acts perpendicular to the shoe sole during speed skating, we developed two estimation methods. The first method uses the coordinates of the front and rear ends of the blade and the ankle joint center. The other method uses the coordinates of the front and rear ends of the blade and the knee joint center. We anticipate that the former method would be more suitable for estimating the GRF direction. This assumption was verified by adopting GRFs measured using FP as true values, with data collected in an environment that permitted FP measurements. Accordingly, this study analyzed the on-land skating motions performed by inline speed skaters as the target movement.

## 2 Materials and methods

### 2.1 Data collection

The participants in this study were 16 experienced university ice speed skaters (7 males and 9 females, male; height: 170.3 ± 3.7 cm, weight: 70.3 ± 6.0 kg, age: 20.3 ± 1.7 years, female; height: 160.8 ± 4.6 cm, weight: 58.0 ± 5.1 kg, age: 19.4 ± 1.1 years) with over 10 years of competitive ice speed skating experience. The participants regularly practice inline speed skating as an off-season training exercise. Prior to participating in the experiment, all participants were provided with written and verbal explanations of the purpose, procedures, risks, and voluntary nature of participation. All participants submitted written informed consent forms to participate in the study. The participants underwent the measurements after warming up in the same manner as before their regular training and inline skating practicing. This study was approved by the Ethical Review Committee of the Japan Institute of Sports Sciences (2022-050).

**Figure 1:**
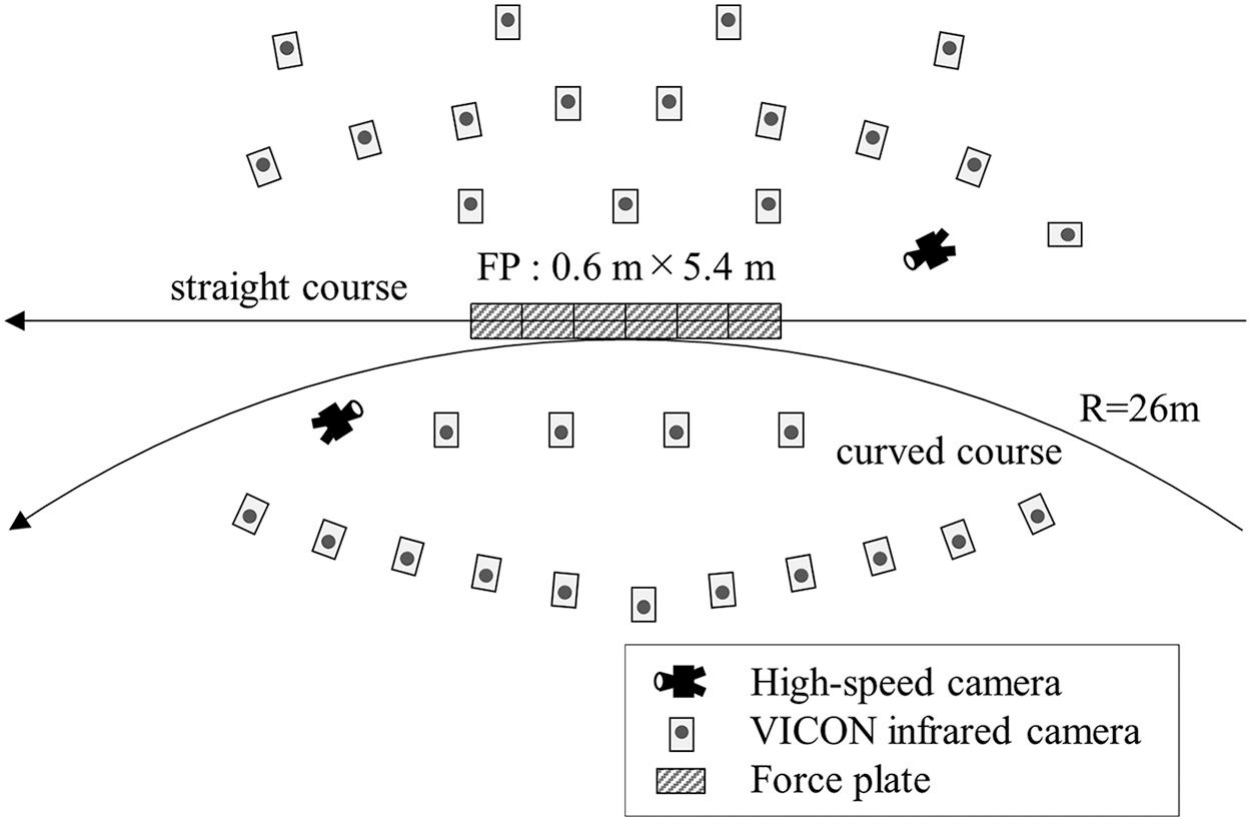
Set up for straight and curved courses. The positions of the high-speed camera and VICON infrared camera are shown. The diagonal-lined part indicates the area where the force plates are embedded in the course (0.6 m wide by 5.4 m long).

The participants performed 10 trials of straight and curve skating using inline speed skates. For both straight and curve skating, the participants completed an approximately 50-meter approach run and passed through the analysis section (Figure 1). The radius of the curved course was set at 26 m to match the radius of the inner lane on the International Skating Union standard C-type track. The 3D coordinates of 47 reflective markers (diameter: 20 mm) attached to the body and inline skates were measured using optical motion capture (Vicon-Vantage, Vicon Motion System Ltd., UK, 300 Hz) to analyze the participants' skating movements. GRFs during skating were measured using an FP (9287B, Kistler, Switzerland, 900 Hz) embedded in the course, measuring 0.6 m wide and 5.4 m long. The moments of contact and release of the inline blade were detected from footage captured using a high-speed camera (Phantom VEO 410S, Vision Research, USA, 300 Hz).

### 2.2 Data analysis

The three-dimensional coordinates of the 47 analysis points obtained via optical motion capture were smoothed using a fourth-order Butterworth low-pass digital filter with a cut-off frequency of 9.0–15.0 Hz, as determined using a residual method (24). The coordinates of the body's center of mass (CM) were calculated using body segment inertia parameters for male and female speed skaters provided by Yokozawa et al. (25). A local coordinate system was set such that the horizontal component of the CM velocity was the Y'-axis, the vertically upward direction was the Z'-axis, and the cross product of the Y' and Z' axes was the X'-axis. The CM velocities during the trials in this study were 9.79 ± 3.34 m/s for straight skating and 8.87 ± 3.87 m/s for curve skating.

**Figure 2:**
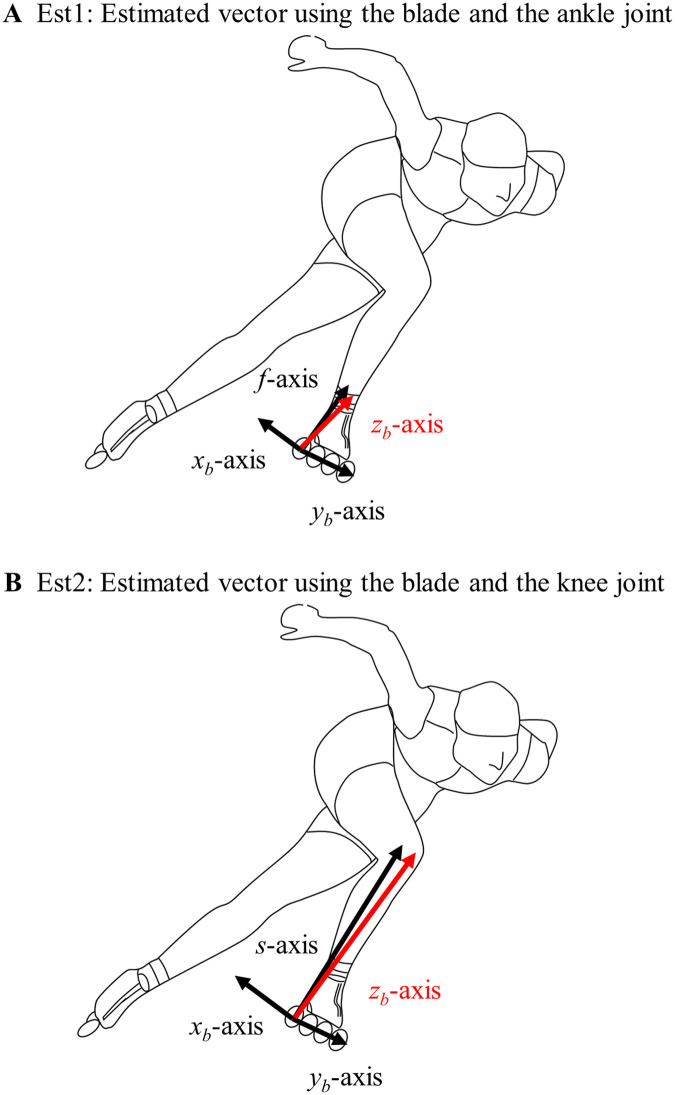
Definition of two methods for estimating the direction of ground reaction forces from the posture of the supporting leg. **(A)** Estimated vector using the blade and the ankle joint, and **(B)** Estimated vector using the blade and the knee joint. The magnitude of each ground reaction force component was estimated by multiplying the resultant force (∑) detected by FP by each component of the unit vector *z_b_* calculated in Est1 and Est2.

Within the local coordinate system, the GRF's direction was estimated from the posture of the supporting leg. Two estimation methods were defined as follows (Figure 2): one using the coordinates of the front and rear ends of the blade and the ankle joint center (hereafter Est1), and the other using the coordinates of the front and rear ends of the blade and the knee joint center (hereafter Est2).

1. Est1: Estimated vector using the blade and the ankle joint

The vector from the rear end of the blade to its front end as the *y_b_*-axis, the vector from the rear end of the blade to the ankle joint as the *f*-axis, and the cross-product of *y_b_* and *f* as the *x_b_*-axis. Vector *z_b_*, which is the cross product of *y_b_* and *x_b_*, was used as the estimated vector (Figure 2A).

(1) Est2: Estimated vector using the blade and the knee joint

The vector from the rear end of the blade to its front end as the *y_b_*-axis, the vector from the rear end of the blade to the knee joint as the *s*-axis, and the cross-product of *y_b_* and *s* as the *x_b_*-axis. Vector *z_b_*, which is the cross product of *y_b_* and *x_b_*, was used as the estimated vector (Figure 2B).

The magnitude of each GRF component was estimated by multiplying the resultant force (∑) detected by the FP by each component of the unit vector *z_b_* calculated in Est1 and Est2.

$$\left[\begin{array}{c} F_{\mathrm{est}X^{\prime }}\\ F_{\mathrm{est}Y^{\prime }}\\ F_{\mathrm{est}Z^{\prime }} \end{array}\right]=F_{\sum }\left[\begin{array}{c} e_{X^{\prime }}\\ e_{Y^{\prime }}\\ e_{Z^{\prime }} \end{array}\right]$$

Here, *e*_X'_, *e*_Y’_, and *e*_Z'_ represent the X', Y', and Z' components, respectively, of the unit vector *z_b_* calculated in Est1 and Est2. *F*_∑_ represents the resultant force of the GRF detected by the FP. *F*_estX'_, *F*_estY'_, and *F*_estZ'_ represent the estimated X', Y', and Z' components of the GRF, respectively. In this study, the magnitude of the GRF (*N*) was expressed as a value divided by the body weight (*N*), yielding a dimensionless quantity.

Since straight skating is a bilaterally symmetrical motion, the data for the left leg were processed by multiplying the X' component value by −1 to reverse left and right, and treated as data for the right leg. Curve skating involves bilaterally asymmetrical motion (26); therefore, data from the left and right legs were analyzed separately. Data for the straight skating motion, right leg during the curve skating motion (curve-right), and left leg during the curve skating motion (curve-left) are shown.

The period during which the leg on the side being analyzed was the supporting leg was divided into contact, single-support, and push-off phases (27, 28). The contact phase is defined as the period from the analyzed side blade contact to one frame before the opposite side blade is turned off. The single-support phase is defined as the period from opposite side blade-off to one frame before the opposite side blade contact. The push-off phase is defined as the period from the opposite side blade-on to one frame before the analyzed side blade-off. The contact and push-off phases are phases during which both legs provide support. The times required for each phase of each skating motion performed in this study are listed in Table 1. Based on this and prior research (27–31), the time-series data were normalized and presented such that the contact and push-off phases each accounted for 20%, and the single-support phase accounted for 60%. The time-series data were normalized using cubic spline interpolation to obtain equally spaced data points (24).

**Table 1:** Time required for the contact phase, single-support phase, and push-off phase of the straight skating, the right leg during curve skating, and the left leg during curve skating (mean ± standard deviation).

Skating motion	Unit	Contact	Single-support	Push-off
		mean ± S.D.	mean ± S.D.	mean ± S.D.
Straight	(s)	0.16 ± 0.06	0.60 ± 0.17	0.16 ± 0.06
	(%)	17.63	64.74	17.63
Curve-right	(s)	0.15 ± 0.06	0.48 ± 0.10	0.14 ± 0.07
	(%)	19.12	62.59	18.28
Curve-left	(s)	0.14 ± 0.07	0.56 ± 0.14	0.15 ± 0.06
	(%)	16.38	66.50	17.13

**Figure 3:**
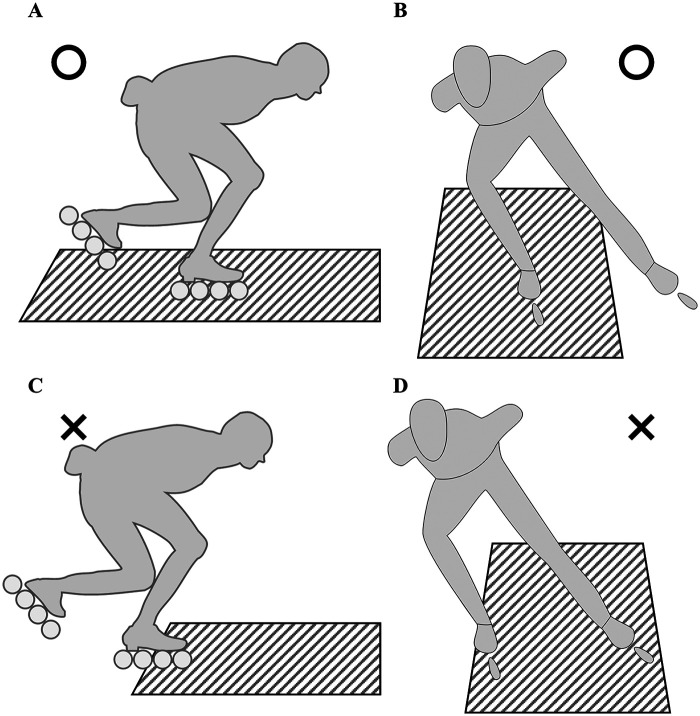
Examples of frames to be analyzed and frames to be excluded from analysis. The diagonal-lined part indicates the area where the force plates are embedded in the course. In this study, frames in which only the analyzed side blade was in contact with the FP were selected for analysis to accurately evaluate the GRFs acting on a single blade **(A)**. For double support, frames in which only the analyzed side blade was in contact with the FP, and the contralateral blade was in contact outside the FP measurement range, were selected for analysis **(B)**. Frames where part of the analyzed side blade was in contact outside the FP measurement range **(C)**, and frames where both blades were simultaneously in contact with the FP **(D)** were excluded from the analysis.

In this study, frames in which only the analyzed side blade was in contact with the FP were selected for analysis to accurately evaluate the GRFs acting on a single blade (Figure 3A). For double support, frames in which only the analyzed side blade was in contact with the FP, and the contralateral blade was in contact outside the FP measurement range, were selected for analysis (Figure 3B). Frames where part of the analyzed side blade was in contact outside the FP measurement range (Figure 3C), and frames where both blades were simultaneously in contact with the FP (Figure 3D) were excluded from the analysis. Therefore, the part of the time-series data normalized for each phase that met the above conditions was used as the frame for analysis. Therefore, the sample size varies at each 1% interval of normalized time. Table 2 shows the number of trials containing the analyzed frames for each participant.

**Table 2:** Number of trials that included the analyzed frames for each participant.

Participant	Straight	Curve-right	Curve-left
A	7	6	7
B	7	6	8
C	5	10	10
D	5	10	10
E	5	3	8
F	7	7	6
G	7	8	8
H	8	5	6
I	9	5	6
J	7	6	6
K	3	6	7
L	9	6	7
M	5	4	8
N	5	10	9
O	5	9	8
P	5	5	10

### 2.3 Statistical analysis

In this study, the magnitudes of each GRF component measured using FP were considered true values, whereas the magnitudes estimated using Est1 and Est2 were considered estimated values. The root mean square error (RMSE) between the time series data of the true values and each estimated value was calculated, with smaller values indicating a lower error.

Furthermore, for estimation methods deemed capable of producing smaller errors based on the RMSE results, verification was carried out at 10% intervals over a normalized time of 5%–95%, as described below. The following verification was conducted on an exploratory basis. First, the slope and intercept of the regression equation were calculated using the true values on the vertical axis and estimated values on the horizontal axis. Next, the intraclass correlation coefficient (ICC, 2.1) analysis was employed to examine the level of agreement between the true and estimated values. In this study, following the guidelines presented by Koo & Li (32) (<0.5: poor, 0.5–0.75: moderate, 0.75–0.9: good, >0.9: excellent), agreement was considered present when the ICC value was 0.5 (moderate) or higher, in accordance with previous studies (33–35). Furthermore, the mean difference between the estimated and true values was calculated as the bias, and the standard deviation was calculated as the precision. Bland-Altman analysis was performed to investigating the presence of systematic bias (36). The 95% limits of agreement (LoA) were calculated as bias ± 1.96 × precision. The 95% confidence interval (95% CI) of the bias was calculated to assess the presence of fixed bias. Fixed bias was considered present when the 95% CI of the bias did not include zero (37). A Bland-Altman plot was created by plotting the mean of both methods on the horizontal axis and the difference between the estimated and true value on the vertical axis. If the correlation between these points was significant, a proportional error was considered present (38). Additionally, considering repeated measurements, the slope and confidence intervals were calculated. The significance level was set at less than 5%. For each 10% interval at which bias was calculated, if the absolute value of the bias was 0.08 or greater at any time point (i.e., a bias of 8% or more of body weight was present), a correction method using a regression equation between the true value and the estimated values was proposed for that skating motion (15, 18, 21, 22).

## 3 Results

### 3.1 Comparison of estimation methods

**Figure 4:**
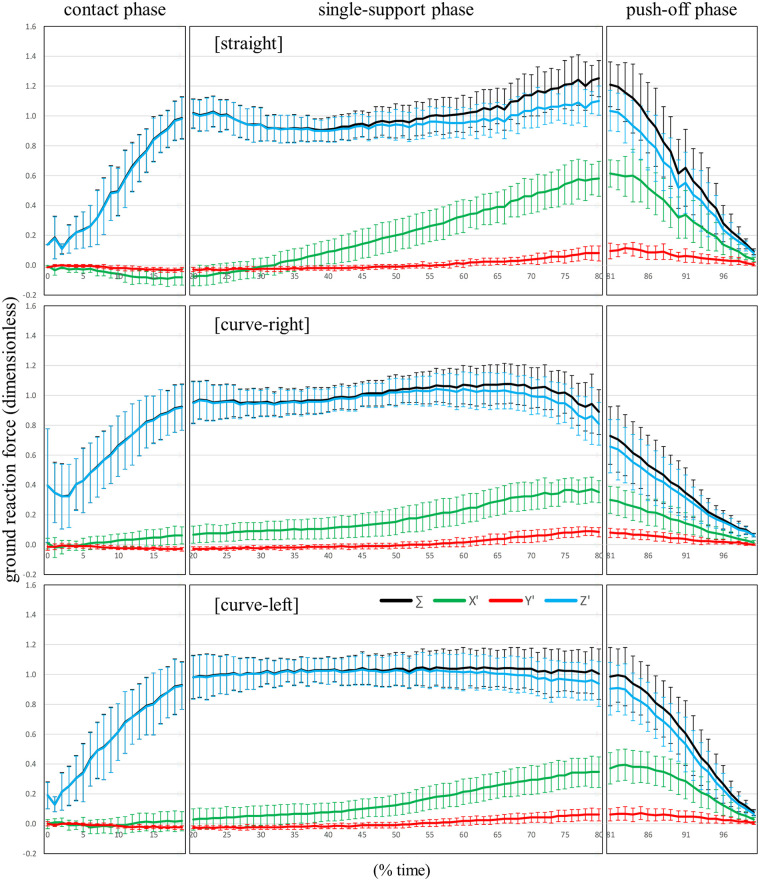
The patterns of the resultant force (∑), X’, Y’, and Z’ components (mean ± standard deviation) of the ground reaction force divided by body weight for the straight skating, the right-leg during curve skating, and the left-leg during curve skating, calculated by FP.

**Figure 5:**
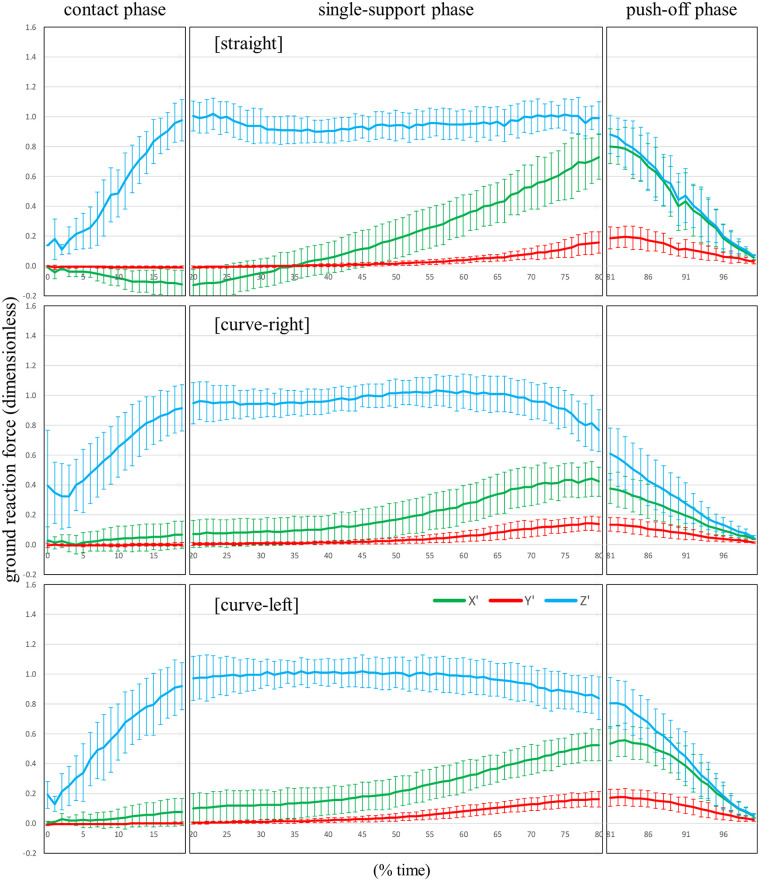
The patterns of the X’, Y’, and Z’ components (mean ± standard deviation) of the ground reaction force divided by body weight for the straight skating, the right leg during curve skating, and the left leg during curve skating, estimated using Est1.

**Figure 6:**
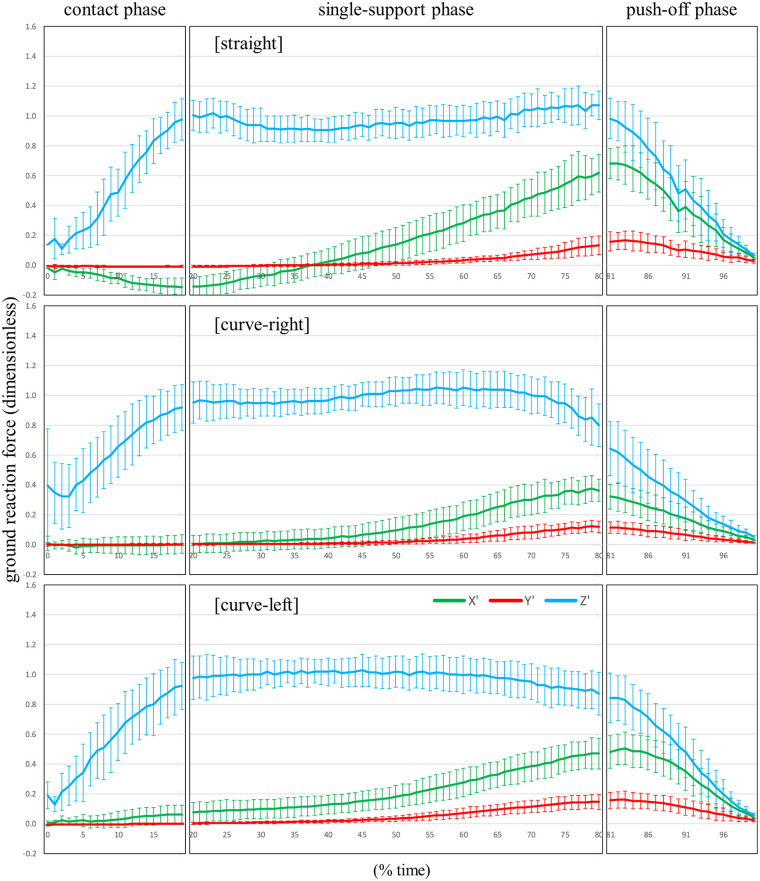
The patterns of the X’, Y’, and Z’ components (mean ± standard deviation) of the ground reaction force divided by body weight for the straight skating, the right leg during curve skating, and the left leg during curve skating, estimated using Est2.

**Figure 7:**
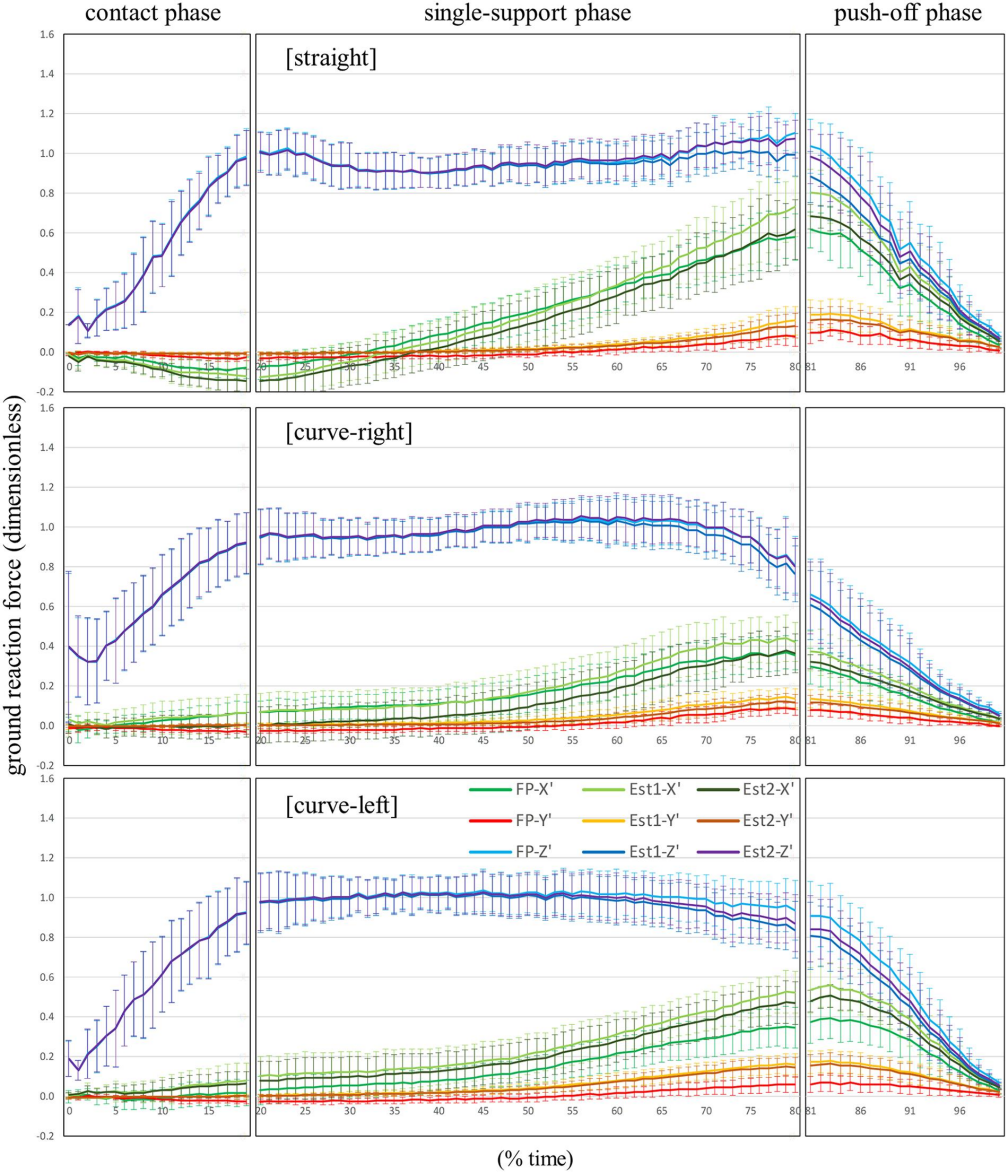
Comparison of the averaged patterns of the X’, Y’, and Z’ components of the ground reaction force divided by body weight for the straight skating, the right leg during curve skating, and the left leg during curve skating, calculated by FP, Est1, and Est2, respectively.

Supplementary Figures S1–S3 show the patterns of the X', Y', and Z' components (mean ± standard deviation) for the unit vector of the true GRF detected by FP, and the unit vector of *z_b_* calculated by Est1 and Est2, respectively, during straight skating, right leg during curve skating, and left leg curve skating. Figures 4–6 show the patterns of the X', Y', and Z' components (mean ± standard deviation) of the GRF divided by body weight for straight skating, right leg during curve skating, and left leg curve skating, calculated using FP, Est1, and Est2, respectively. Figure 7 compares the averaged patterns of the X', Y', and Z' components of the GRF divided by body weight for straight skating, the right leg during curve skating, and the left leg during curve skating, calculated using FP, Est1, and Est2, respectively. The GRF components calculated by Est1 and Est2 showed overall waveforms, phases of increase/decrease, and overall force magnitudes similar to those of the FP. Table 3 shows the RMSE between the true values (FP) and the estimated values (Est1 and 2). Among these, the estimation of the X' component for the left leg during curve skating using Est1 showed a relatively large RMSE, but it was only slightly above 0.1 (0.109). In other words, the force error was within a range equivalent to approximately 10% of the body weight. Est2 yielded smaller values than Est1 for all components across all skating motions. Based on the RMSE results, Est2 was determined to be the superior estimation method, and verification was performed at a 10% time interval.

**Table 3:** The RMSE between the true values (FP) and the estimated values (Est1, 2) for the X’, Y’, and Z’ components of the ground reaction force for the straight skating, the right leg during curve skating, and the left leg during curve skating.

Estimation method	Component	Straight	Curve-right	Curve-left
X’	Est1	0.078	0.080	0.109
	Est2	0.059	0.074	0.074
Y’	Est1	0.036	0.045	0.062
	Est2	0.030	0.040	0.054
Z’	Est1	0.040	0.022	0.046
	Est2	0.017	0.010	0.030

The magnitude of the force (*N*) was expressed as a value divided by the body weight (*N*).

### 3.2 Detailed verification of Est2

Supplementary Tables S1–S3 present the sample size, mean ± standard deviation, regression slope and intercept between true and estimated values, ICC, bias, precision, and Bland-Altman analysis results for the X', Y', and Z' components of the GRF at each 10% time interval for the straight skating, the right-leg during curve skating, and the left-leg during curve skating, calculated using FP and Est2. The unit of sample size was a trial. For the straight skating, the ICC results indicated that the X' and Z' components were in agreement with the true values at all time points (Supplementary Table S1). The Y' component was in agreement at 65%–85% of the time points. For the Y' component at the 5%–55% and 95% time points, where agreement was not achieved, both fixed and proportional errors were present. For the right leg during curve skating, the ICC results indicated that the X' and Z' components were in agreement with the true values at all time points (Supplementary Table S2). The Y' component was in agreement at 65%–85% of the time points. For the Y' component at the 5%–55% time points, where agreement was not achieved, both fixed and proportional errors were present. Similarly, for the Y' component at the 95% time point, which was not in agreement, a fixed error occurred. For the left leg during curve skating, the ICC results indicated that the X' and Z' components were in agreement with the true values at all time points (Supplementary Table S3). The Y' component was in agreement at the 95% time point. For the Y' component at 5%–85% of the time points, where agreement was not achieved, a fixed error was present in all cases. Additionally, proportional error was present for the Y' component at the 5%–35% and 65%–85% time points. For the X' and Y' components at the 65% and 75% points of the left leg during curve skating, the absolute value of the bias was 0.08 or greater (Supplementary Table S3). We propose a correction method using the regression equation between the true and estimated values for the left leg during curve skating because a bias larger than the criterion set in this study was confirmed.

### 3.3 Correction for the left leg during curve skating

A single regression analysis was performed on the true and estimated values for all phases of the left leg during curve skating. The results yielded the following regression equations for the X', Y', and Z' components:

$$FP_{X^{\prime }}=\;0.847\;\times \;E_{X^{\prime }}-\;0.029$$

$$FP_{Y^{\prime }}=\;0.596\;\times \;E_{Y^{\prime }}-\;0.025$$

$$FP_{Z^{\prime }}=\;0.987\;\times \;E_{Z^{\prime }}+\;0.030$$

Here, FP_X'_, FP_Y'_, and FP_Z'_ represent the magnitudes of the GRF components X', Y', and Z' detected by FP, whereas *E*_X'_, *E*_Y'_, and *E*_Z'_ represent the magnitudes of the GRF components X', Y’, and Z' estimated using Est2. Using the regression equations above, corrections were applied by substituting the Est2 components at each time point into *E*_X'_, *E*_Y’_, and *E*_Z'_. The RMSE between the true values (FP) and the corrected estimated values (hereafter, Est2C) was 0.033 for the X' component, 0.017 for the Y' component, and 0.023 for the Z' component. All RMSE values were smaller than those before the correction. Supplementary Table S4 shows the verification results of each component of the GRF for the left leg during curve skating, calculated using FP and Est2C, similar to Supplementary Table S3. One participant's data was excluded, and out-of-sample validation was performed. The ICC results indicated that the X' and Z' components were in agreement with the true values at all time points. The Y' component was in agreement at the 55%–95% time points. For the Y' component at 5%–45% time points, where agreement was not achieved, a proportional error was present in all cases. No bias greater than 0.08 was observed for any component at any time point.

## 4 Discussion

### 4.1 Regarding the feasibility of estimation and comparison of the two estimation methods

The objective of this study is to propose and verify a method for estimating the GRF direction from the posture of the supporting leg during speed skating. For both Est1 and Est2, the RMSE relative to FP was within approximately 0.1 times that body weight (Table 3). Therefore, the hypothesis that “the direction of GRFs can be accurately estimated from the posture of the supporting leg” was accepted. Fluit et al. (15) estimated the GRFs during activities of daily living from optical motion capture data, reported an RMSE of 0.097 for the vertical component during stair ascent, and concluded that this estimation was feasible. Similarly, Komaris et al. (18) estimated the GRFs during running from optical motion capture data, reported an RMSE of 0.134 for the vertical component, and concluded that this estimation was feasible. Furthermore, Jiang et al. (22) estimated GRFs during walking from IMU sensor data, reported an RMSE of 0.10 for the vertical component, and concluded that estimation was feasible. Furthermore, Lichtwark et al. (21) estimated the GRFs during running from marker-less motion capture data, reported an RMSE of 0.188 for the vertical component, and recommended this estimation method. They also illustrated that the RMSE for the vertical and lateral components during the cutting maneuver was 0.100 or higher. The RMSE values in this study were comparable to those reported in previous studies that estimated the GRFs during activities such as walking, stair ascent, cutting, and running. This suggests that both estimation methods, Est1 and Est2, were fundamentally capable of reasonably estimating each component of the GRF for each skating motion.

Estimation method 1 used the blade and ankle joints, whereas Estimation method 2 used the blade and knee joints. Comparing the RMSE of the two methods, Est2 showed smaller errors than Est1 for all components across all skating motions (Table 3). Therefore, the hypothesis that “the estimation method using the coordinates of the blade's front/rear ends and ankle joint center is more suitable for estimating the GRF direction” was not supported by the results. The estimation method using the coordinates of the front and rear ends of the blade and the knee joint center was more suitable for estimating the GRFs. Anatomically, due to ankle joint mobility (dorsiflexion, plantar flexion, pronation, and supination), the estimation method using the ankle joint is thought to reproduce the direction of the forces better applied perpendicular to the sole.

Nevertheless, the reason why Est2, using the knee joint, had smaller errors than Est1 was likely that the vector length used for the estimation was longer. The distance from the rear end of the blade to the knee joint was greater than that from the rear end of the blade to the ankle joint (Figure 2). Although errors in calculating the coordinate positions of reflective markers via motion capture affect vector orientation, the impact of this error diminishes as the vector length increases (39). Est2 used longer vectors for estimation than Est1, resulting in less influence from motion capture-dependent errors. Consequently, the estimated value is considered to be more stable, leading to smaller errors relative to the true values.

### 4.2 Estimation of each component by Est2

The bias values of both straight skating and the right leg during curve skating for each component were below the 0.08 threshold set based on previous studies at all time points (Supplementary Tables S1 and S2). Furthermore, the ICC results indicated that the X' and Z' components were in agreement with the true values at all time points. Using Est2, it was possible to accurately estimate values close to the true values for the X' and Z' components of both straight skating and the right leg during curve skating. However, several time points were identified where the Y' component did not agree with the true value. The estimation of the Y' component can be considered less favorable compared to the X' and Z' components. Caution is required when using Est2 to investigate the skater's propulsive force (Y' component) during skating. Nevertheless, the magnitude of the Y' component was very small, ranging from 0 to 0.1 times × body weight (Figure 4: upper and middle rows, red line). The bias in the Y’ component was greatest at the 85% time point of straight skating, amounting to 0.06 times body weight. When calculating lower limb joint torque, the moment arm to the hip joint center is at its maximum length. During speed skating, the moment arm from the COP to the hip joint center is approximately 0.5 m, and calculations show that the moment error caused by the Y’ component error is approximately 0.29 Nm/kg. The hip flexion-extension moment during speed skating varies within a range of approximately 3.0 Nm/kg (3), so the error has less than a 10% impact on this magnitude. The bias in the Y' component outside of the 85% time point of the straight skating phase ranges from 0.00 to 0.04 times body weight, which makes its impact even smaller. Furthermore, the hip internal and external rotation torques during skating are very small (3). Additionally, the Y' component is thought to have no effect on adduction and abduction torques. Therefore, its impact on calculations such as the joint torque or joint power of the supporting leg is minimal, making Est2 a useful choice.

For the left leg during curve skating, at the 75% and 85% time points—roughly around the time the right blade-on—the bias for both the X' and Y' component was 0.08 or greater (Supplementary Table S3). Looking at the 95% CI of the bias at 75% and 85% time points (Supplementary Table S3), as well as at the time-series graph of GRFs (average value) for the left leg during curve skating calculated by FP and Est2 (Figure 7, bottom), Est2 overestimated the X' and Y' components and underestimated the Z' component. As the push-off phase progresses, the left shank becomes more inclined (29, 31), and it is thought that Est2 reflected this inclination of the left shank and estimated the Z' component to be smaller than the true value. However, the actual GRF direction detected by the FP was not as inclined as that of the left shank and remained more vertical until the left blade was off. One reason for the underestimation of the Z' component in Est2 is that it did not take into account the force pressing vertically against the left inner wall of the inclined left shoe due to the outward tilt of the left shank (29, 31) near the push-off phase of the left leg during curve skating. Applying corrections using regression equations reduced the bias, which was initially 0.08 or higher, to 0.01 (X' component, 75% time point), 0.00 (X' component, 85% time point), 0.00 (Y' component, 75% time point), and 0.00 (Y' component, 85% time point) (Supplementary Table S4). After correction, the left leg achieved accuracy equivalent to or better than that of the right leg during curve skating. Even after correction, the ICC results indicated that the X' and Z' components were in agreement with the true values at all time points. Regarding the Y' component, even after correction, some time points still showed no agreement according to the ICC results; therefore, caution is required when examining the skater's propulsive force. Nevertheless, similar to straight skating and the right leg during curve skating, Est2C is useful for calculating the joint torques and joint powers of the left leg during curve skating.

### 4.3 Limitations

This study had several limitations. First, the target movement analyzed in this study involved inline speed skating motion performed on land. The results of this study cannot be assumed to be directly transferable to ice conditions because the friction coefficients of the gliding surfaces differ between inline and ice speed skating. More rigorous verification would require reaction forces measured directly on ice with special instrumented blades equipped with force sensors, as the true values for comparison with the estimated values. Nevertheless, the skating motion patterns of inline and ice speed skaters are fundamentally similar (40, 41). Accordingly, the fundamental findings of this study, particularly those related to estimation of the X' and Z' components, are likely applicable to ice speed skating. Second, the skating motions performed near the centers of the straight and curved track sections were analyzed in this study. As a result, skating motions occurring at transition points between straight and curved sections, as well as the starting motion, were not evaluated. Verification of these additional sections is necessary to estimate reaction forces across the entire skating track.

## 5 Conclusion

This study aimed to propose and validate a method for estimating GRF direction from the posture of the supporting leg during speed skating. The results supported the hypothesis that GRF direction can be accurately estimated from the posture of the supporting leg. In contrast, the hypothesis that “the estimation method using the coordinates of the blade's front/rear ends and ankle joint center is more suitable for estimating the GRFs direction” was not supported by the results. The estimation method using the coordinates of the blade's front and rear ends and the knee joint center yielded a smaller RMSE relative to the true values than the method that using the ankle joint center, indicating greater suitability for estimation.

Detailed verification of the estimation method using the coordinates of the blade's front and rear ends and the knee joint center showed that, during straight skating and for the right leg during curve skating, the bias for each component remained below the predefined criterion of 8% of body weight at all time points. For the left leg during curve skating, application of a regression-based correction also reduced the bias. Furthermore, the ICC analysis indicated agreement between the true and estimated values for the X' and Z' components across all skating motions. For the Y' component, several time points showed no agreement with the true values. Caution is required when using this estimation method to evaluate a skater's propulsive force (Y' component) during skating. However, because the magnitude of the Y' component is very small (0–0.1 times body weight), its influence on calculations such as the joint torque or joint power of the supporting leg is minimal. Therefore, this estimation method is considered useful for biomechanical analysis of speed skating. When used for actual ice speed skating, it is possible to estimate the reaction forces of the ice during skating motions by combining devices that measure resultant forces, such as foot-pressure sensors, with the reaction force direction estimation method introduced in this study.

## Data Availability

The raw data supporting the conclusions of this article will be made available by the authors, without undue reservation.
