# Supplementary material for: Estimation of ground reaction force direction in inline speed skating motion
Source: Front Sports Act Living. 2026 Apr 13;8:1782354. doi: 10.3389/fspor.2026.1782354 (PMC13111381; doi:10.3389/fspor.2026.1782354)
Supplement: Supplementary file 1 [file Datasheet1.pdf]

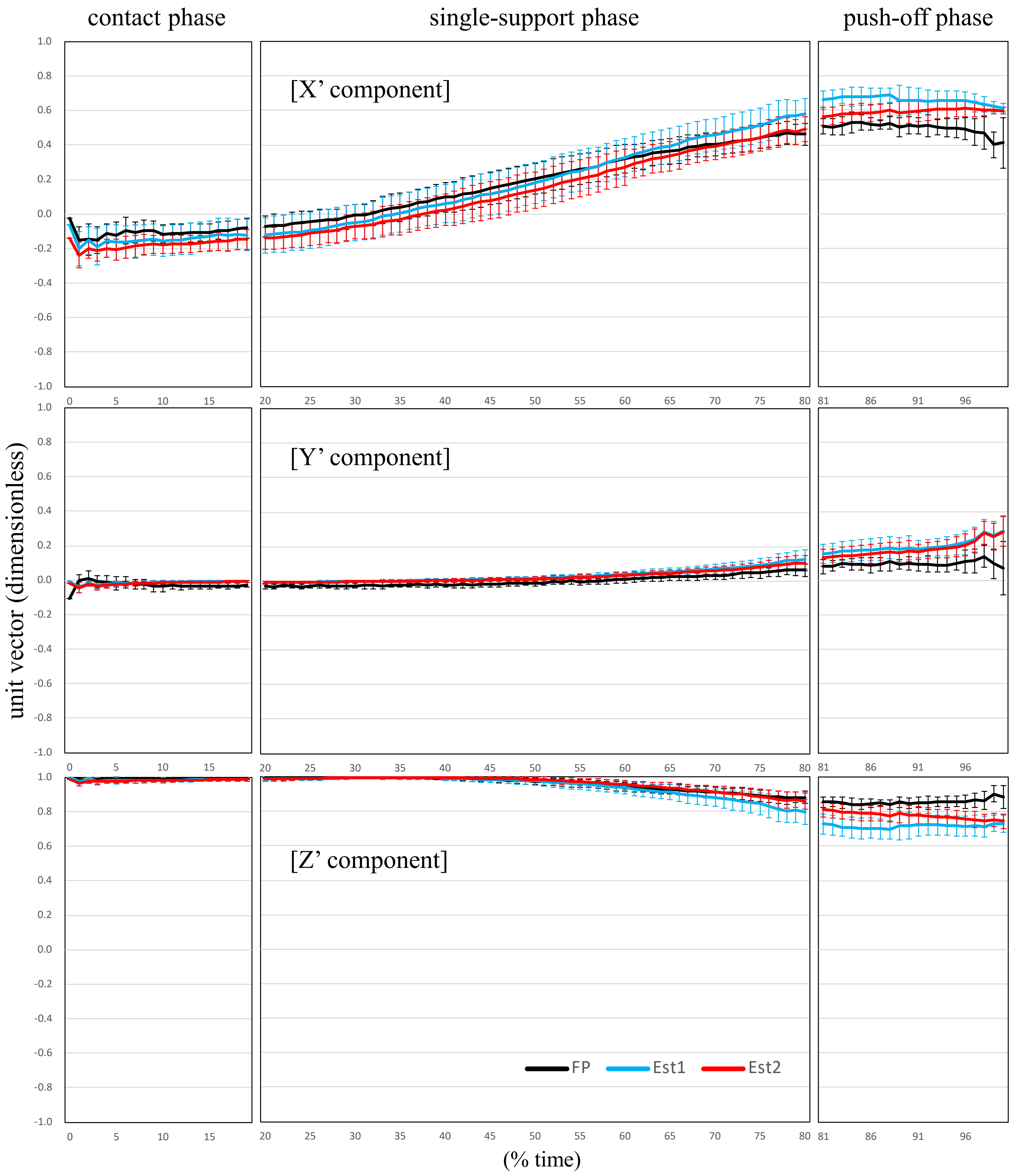

Figure S1

The patterns of the X', Y', and Z' components (mean ± standard deviation) for the unit vector of the true GRF detected by FP, and the unit vector of *zb* calculated by Est1 and Est2, for straight skating.

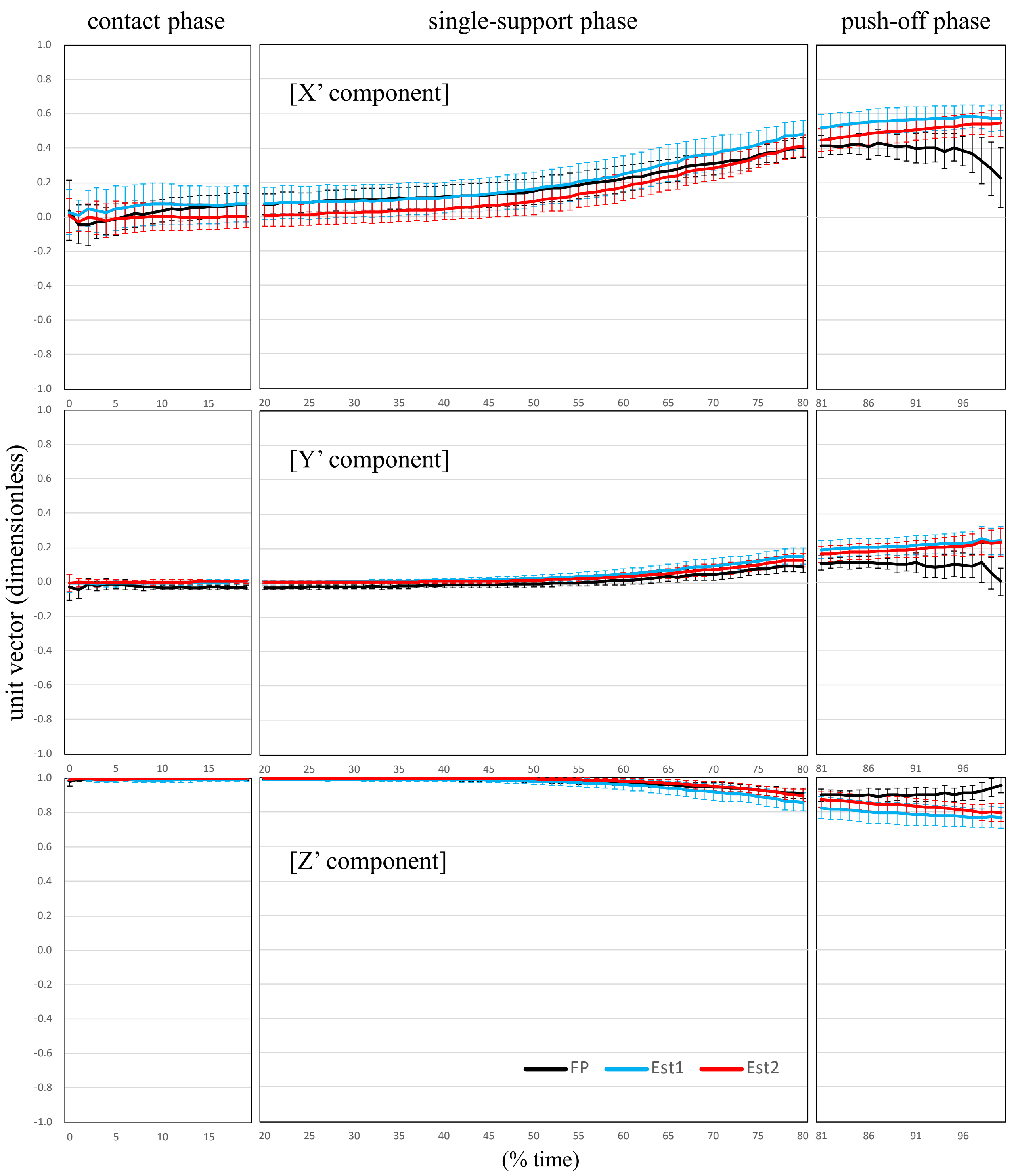

Figure S2

The patterns of the X', Y', and Z' components (mean ± standard deviation) for the unit vector of the true GRF detected by FP, and the unit vector of *zb* calculated by Est1 and Est2, for right leg during curve skating.

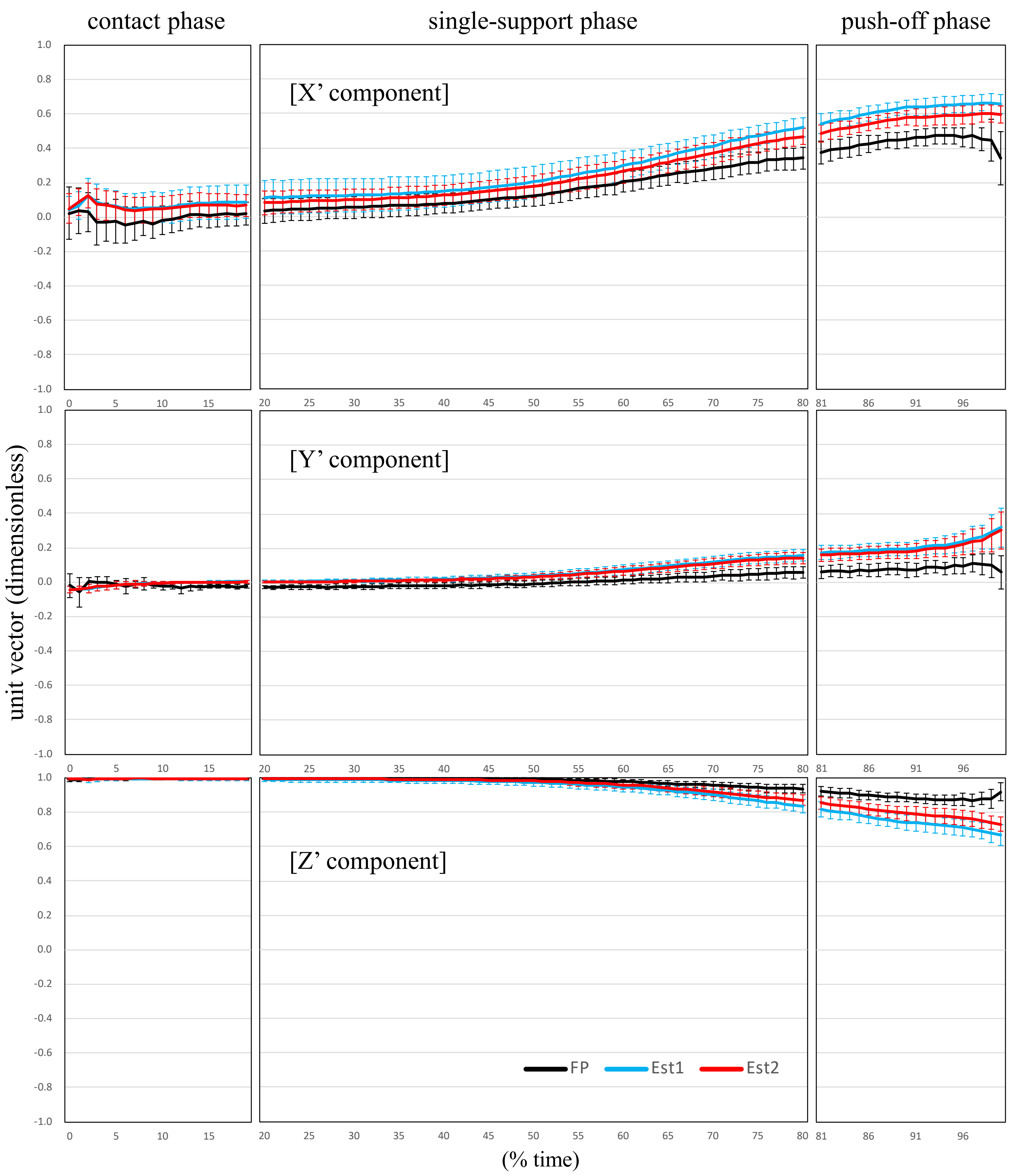

Figure S3
The patterns of the X', Y', and Z' components (mean $\pm$ standard deviation) for the unit vector of the true GRF detected by FP, and the unit vector of *zb* calculated by Est1 and Est2, for left leg during curve skating.

Table S1
The sample size, mean ± standard deviation, regression slope and intercept between true and estimated values, ICC, bias, precision, and Bland-Altman analysis results for the X', Y', and Z' components of ground reaction force at each 10% interval time points for the straight skating, calculated using FP and Est2. The magnitude of the force (N) was expressed as a value divided by the body weight (N).

	phase	% time		n (trial)	mean ± S.D. (no unit)	slope	intercept	ICC (2.1)		95%CI	Bland-Altman analysis: bias ± precision	LoA	95%CI of bias	Bland-Altman plot: slope	r	*p*		95%CI
X'	contact	5%	FP	19	-0.03 ± 0.02													
			Est2	19	-0.05 ± 0.02	0.63	0.00	0.54	†	-0.05 ~ 0.82	-0.02 ± 0.02	0.02 ~ -0.05	-0.01 ~ -0.03	0.22	0.18	0.460	n.s.	-0.30 ~ 0.58
		15%	FP	36	-0.09 ± 0.04													
			Est2	36	-0.13 ± 0.05	0.77	0.02	0.54	†	-0.10 ~ 0.83	-0.05 ± 0.03	0.01 ~ -0.10	-0.04 ~ -0.05	0.14	0.09	0.608	n.s.	-0.25 ~ 0.40
	single-support	25%	FP	39	-0.05 ± 0.06													
			Est2	39	-0.12 ± 0.07	0.79	0.04	0.60	†	-0.08 ~ 0.87	-0.07 ± 0.03	0.00 ~ -0.13	-0.06 ~ -0.08	0.44	0.23	0.164	n.s.	-0.10 ~ 0.50
		35%	FP	54	0.03 ± 0.08													
			Est2	54	-0.03 ± 0.08	0.87	0.06	0.72	†	-0.06 ~ 0.92	-0.06 ± 0.03	-0.01 ~ -0.12	-0.06 ~ -0.07	0.73	0.25	0.069	n.s.	-0.02 ~ 0.48
		45%	FP	62	0.15 ± 0.10													
			Est2	62	0.08 ± 0.11	0.89	0.07	0.81	††	-0.04 ~ 0.95	-0.06 ± 0.02	-0.02 ~ -0.11	-0.06 ~ -0.07	1.52	0.37	0.003	**	0.13 ~ 0.57
		55%	FP	58	0.26 ± 0.11													
			Est2	58	0.21 ± 0.12	0.86	0.08	0.88	††	0.00 ~ 0.97	-0.05 ± 0.03	0.00 ~ -0.11	-0.04 ~ -0.06	2.05	0.52	0.000	***	0.30 ~ 0.69
		65%	FP	48	0.39 ± 0.10													
			Est2	48	0.36 ± 0.11	0.88	0.07	0.94	†††	0.48 ~ 0.98	-0.03 ± 0.02	0.02 ~ -0.08	-0.02 ~ -0.04	1.97	0.46	0.001	**	0.20 ~ 0.65
		75%	FP	30	0.54 ± 0.13													
			Est2	30	0.55 ± 0.15	0.82	0.09	0.97	†††	0.94 ~ 0.99	0.00 ± 0.04	0.07 ~ -0.07	0.02 ~ -0.01	2.88	0.72	0.000	***	0.47 ~ 0.85
	push-off	85%	FP	11	0.57 ± 0.14													
			Est2	11	0.62 ± 0.13	1.02	-0.07	0.90	†††	0.03 ~ 0.98	0.05 ± 0.03	0.12 ~ -0.01	0.07 ~ 0.04	-0.89	-0.21	0.529	n.s.	-0.72 ~ 0.45
		95%	FP	15	0.19 ± 0.09													
			Est2	15	0.23 ± 0.11	0.86	-0.01	0.91	†††	-0.01 ~ 0.98	0.04 ± 0.02	0.08 ~ 0.00	0.05 ~ 0.03	3.53	0.69	0.004	**	0.26 ~ 0.88
Y'	contact	5%	FP	19	-0.01 ± 0.01													
			Est2	19	-0.01 ± 0.00	1.22	0.00	0.38		-0.09 ~ 0.71	0.00 ± 0.01	0.02 ~ -0.02	0.00 ~ 0.00	-0.55	-0.75	0.000	***	-0.89 ~ -0.43
		15%	FP	36	-0.03 ± 0.01													
			Est2	36	-0.01 ± 0.01	0.23	-0.03	0.03		-0.08 ~ 0.19	0.02 ± 0.01	0.05 ~ -0.01	0.02 ~ 0.01	-0.35	-0.64	0.000	***	-0.79 ~ -0.38
	single-support	25%	FP	39	-0.03 ± 0.01													
			Est2	39	-0.01 ± 0.01	0.16	-0.03	0.02		-0.07 ~ 0.16	0.02 ± 0.01	0.05 ~ -0.01	0.03 ~ 0.02	-0.30	-0.55	0.000	***	-0.74 ~ -0.28
		35%	FP	54	-0.02 ± 0.01													
			Est2	54	0.00 ± 0.01	0.20	-0.02	0.03		-0.06 ~ 0.17	0.02 ± 0.01	0.04 ~ -0.01	0.02 ~ 0.02	-0.29	-0.54	0.000	***	-0.70 ~ -0.31
		45%	FP	62	-0.02 ± 0.02													
			Est2	62	0.01 ± 0.01	0.55	-0.02	0.14		-0.08 ~ 0.39	0.02 ± 0.01	0.05 ~ 0.00	0.03 ~ 0.02	-0.25	-0.34	0.006	**	-0.54 ~ -0.10
		55%	FP	58	0.00 ± 0.02													
			Est2	58	0.02 ± 0.02	0.77	-0.02	0.28		-0.10 ~ 0.61	0.03 ± 0.02	0.06 ~ 0.00	0.03 ~ 0.02	-0.28	-0.29	0.029	*	-0.51 ~ -0.03
		65%	FP	48	0.03 ± 0.02													
			Est2	48	0.05 ± 0.02	0.84	-0.01	0.52	†	-0.09 ~ 0.81	0.02 ± 0.01	0.05 ~ -0.01	0.03 ~ 0.02	-0.18	-0.13	0.370	n.s.	-0.40 ~ 0.16
		75%	FP	30	0.06 ± 0.04													
			Est2	30	0.10 ± 0.05	0.72	-0.01	0.61	†	-0.09 ~ 0.87	0.04 ± 0.03	0.09 ~ -0.01	0.05 ~ 0.03	0.49	0.29	0.119	n.s.	-0.08 ~ 0.59
	push-off	85%	FP	11	0.10 ± 0.04													
			Est2	11	0.16 ± 0.06	0.64	0.00	0.55	†	-0.09 ~ 0.88	0.06 ± 0.03	0.12 ~ 0.00	0.08 ~ 0.04	1.07	0.62	0.042	*	0.00 ~ 0.88
		95%	FP	15	0.04 ± 0.02													
			Est2	15	0.07 ± 0.04	0.47	0.00	0.42		-0.11 ~ 0.79	0.04 ± 0.02	0.08 ~ -0.01	0.05 ~ 0.02	0.89	0.73	0.002	**	0.32 ~ 0.90
Z'	contact	5%	FP	19	0.24 ± 0.12													
			Est2	19	0.23 ± 0.12	1.01	0.00	1.00	†††	0.99 ~ 1.00	0.00 ± 0.00	0.00 ~ -0.01	0.00 ~ 0.00	-20.41	-0.49	0.033	*	-0.77 ~ -0.03
		15%	FP	36	0.84 ± 0.15													
			Est2	36	0.83 ± 0.15	1.00	0.01	1.00	†††	0.95 ~ 1.00	-0.01 ± 0.00	0.00 ~ -0.01	-0.01 ~ -0.01	1.31	0.04	0.830	n.s.	-0.30 ~ 0.36
	single-support	25%	FP	39	1.01 ± 0.10													
			Est2	39	1.00 ± 0.10	1.01	-0.01	1.00	†††	0.98 ~ 1.00	-0.01 ± 0.01	0.01 ~ -0.02	0.00 ~ -0.01	-4.11	-0.25	0.123	n.s.	-0.52 ~ 0.07
		35%	FP	54	0.91 ± 0.09													
			Est2	54	0.91 ± 0.09	1.01	-0.01	1.00	†††	1.00 ~ 1.00	0.00 ± 0.01	0.01 ~ -0.01	0.00 ~ 0.00	-3.00	-0.20	0.148	n.s.	-0.44 ~ 0.07
		45%	FP	62	0.93 ± 0.08													
			Est2	62	0.94 ± 0.08	0.96	0.03	0.99	†††	0.94 ~ 1.00	0.01 ± 0.01	0.02 ~ -0.01	0.01 ~ 0.00	4.49	0.40	0.001	**	0.16 ~ 0.59
		55%	FP	58	0.96 ± 0.10													
			Est2	58	0.97 ± 0.10	0.99	0.00	0.99	†††	0.79 ~ 1.00	0.01 ± 0.01	0.02 ~ 0.00	0.01 ~ 0.01	0.97	0.07	0.597	n.s.	-0.19 ~ 0.32
		65%	FP	48	0.99 ± 0.10													
			Est2	48	1.00 ± 0.10	1.02	-0.03	0.99	†††	0.92 ~ 1.00	0.01 ± 0.01	0.03 ~ -0.01	0.01 ~ 0.01	-2.84	-0.24	0.096	n.s.	-0.49 ~ 0.05
		75%	FP	30	1.08 ± 0.12													
			Est2	30	1.07 ± 0.11	1.10	-0.11	0.98	†††	0.96 ~ 0.99	-0.01 ± 0.02	0.04 ~ -0.05	0.00 ~ -0.01	-3.25	-0.60	0.000	***	-0.79 ~ -0.30
	push-off	85%	FP	11	0.89 ± 0.17													
			Est2	11	0.84 ± 0.18	0.93	0.11	0.96	†††	0.14 ~ 0.99	-0.05 ± 0.03	0.00 ~ -0.10	-0.03 ~ -0.06	3.08	0.45	0.170	n.s.	-0.23 ~ 0.82
		95%	FP	15	0.32 ± 0.14													
			Est2	15	0.29 ± 0.12	1.09	0.01	0.96	†††	0.12 ~ 0.99	-0.04 ± 0.02	0.00 ~ -0.07	-0.03 ~ -0.04	-4.86	-0.67	0.006	**	-0.87 ~ -0.21

†††: ICC > 0.9, ††: ICC > 0.75, †: ICC ≧ 0.5, ***: p < 0.001, **: p < 0.01, *: p < 0.05, n.s.: no significant

Table S2

The sample size, mean ± standard deviation, regression slope and intercept between true and estimated values, ICC, bias, precision, and Bland-Altman analysis results for the X’, Y’, and Z’ components of ground reaction force at each 10% interval time points for the right-leg during curve skating, calculated using FP and Est2. The magnitude of the force (N) was expressed as a value divided by the body weight (N).

	phase	% time		n (trial)	mean ± S.D. (no unit)	slope	intercept	ICC (2.1)		ICC 95%CI	Bland-Altman analysis: bias ± precision	LoA	95%CI of bias	Bland-Altman plot: slope	r	p		95%CI
X'	contact	5%	FP	36	0.00 ± 0.04													
			Est2	36	-0.01 ± 0.05	0.52	0.01	0.61	†	0.36 ~ 0.78	-0.01 ± 0.04	0.07 ~ -0.08	0.00 ~ -0.02	0.25	0.25	0.144	n.s.	-0.09 ~ 0.53
		15%	FP	61	0.05 ± 0.05													
			Est2	61	0.00 ± 0.06	0.85	0.05	0.65	†	-0.07 ~ 0.89	-0.05 ± 0.02	-0.01 ~ -0.10	-0.05 ~ -0.06	0.45	0.19	0.142	n.s.	-0.07 ~ 0.42
	single-support	25%	FP	80	0.08 ± 0.05													
			Est2	80	0.01 ± 0.06	0.84	0.07	0.54	†	-0.05 ~ 0.85	-0.07 ± 0.02	-0.02 ~ -0.11	-0.06 ~ -0.07	0.52	0.22	0.049	*	0.00 ~ 0.42
		35%	FP	83	0.10 ± 0.06													
			Est2	83	0.03 ± 0.06	0.91	0.07	0.58	†	-0.06 ~ 0.86	-0.07 ± 0.03	-0.02 ~ -0.12	-0.06 ~ -0.08	0.00	0.00	0.993	n.s.	-0.22 ~ 0.21
		45%	FP	77	0.13 ± 0.08													
			Est2	77	0.07 ± 0.07	1.02	0.06	0.69	†	-0.06 ~ 0.91	-0.07 ± 0.03	-0.01 ~ -0.12	-0.06 ~ -0.07	-0.62	-0.22	0.058	n.s.	-0.42 ~ 0.01
		55%	FP	69	0.19 ± 0.09													
			Est2	69	0.14 ± 0.09	0.99	0.06	0.80	††	-0.05 ~ 0.94	-0.06 ± 0.03	0.00 ~ -0.11	-0.05 ~ -0.06	-0.36	-0.12	0.332	n.s.	-0.34 ~ 0.12
		65%	FP	55	0.29 ± 0.09													
			Est2	55	0.25 ± 0.10	0.89	0.07	0.90	††	0.11 ~ 0.97	-0.04 ± 0.03	0.01 ~ -0.09	-0.03 ~ -0.04	1.26	0.33	0.013	*	0.07 ~ 0.55
		75%	FP	29	0.37 ± 0.08													
			Est2	29	0.36 ± 0.08	0.89	0.05	0.94	†††	0.88 ~ 0.97	-0.01 ± 0.03	0.04 ~ -0.06	0.00 ~ -0.02	0.68	0.22	0.248	n.s.	-0.16 ~ 0.54
	push-off	85%	FP	18	0.25 ± 0.09													
			Est2	18	0.27 ± 0.09	1.01	-0.03	0.94	†††	0.17 ~ 0.99	0.03 ± 0.02	0.06 ~ -0.01	0.04 ~ 0.02	-0.70	-0.13	0.603	n.s.	-0.56 ~ 0.36
		95%	FP	16	0.08 ± 0.04													
			Est2	16	0.10 ± 0.04	0.96	-0.02	0.83	††	-0.04 ~ 0.96	0.02 ± 0.01	0.04 ~ 0.00	0.03 ~ 0.02	-0.07	-0.02	0.936	n.s.	-0.51 ~ 0.48
Y'	contact	5%	FP	36	-0.01 ± 0.01													
			Est2	36	0.00 ± 0.01	1.11	-0.01	0.28		-0.02 ~ 0.54	0.01 ± 0.01	0.03 ~ -0.02	0.01 ~ 0.00	-0.52	-0.74	0.000	***	-0.86 ~ -0.54
		15%	FP	61	-0.03 ± 0.01													
			Est2	61	0.00 ± 0.01	0.35	-0.03	0.05		-0.04 ~ 0.18	0.03 ± 0.01	0.05 ~ 0.01	0.03 ~ 0.02	-0.20	-0.30	0.017	*	-0.52 ~ -0.05
	single-support	25%	FP	80	-0.02 ± 0.01													
			Est2	80	0.00 ± 0.01	-0.37	-0.02	-0.02		-0.07 ~ 0.05	0.03 ± 0.01	0.06 ~ 0.00	0.03 ~ 0.03	-0.32	-0.72	0.000	***	-0.81 ~ -0.59
		35%	FP	83	-0.02 ± 0.01													
			Est2	83	0.01 ± 0.01	0.58	-0.02	0.05		-0.05 ~ 0.18	0.03 ± 0.01	0.05 ~ 0.00	0.03 ~ 0.02	-0.42	-0.69	0.000	***	-0.79 ~ -0.56
		45%	FP	77	-0.01 ± 0.02													
			Est2	77	0.01 ± 0.01	0.79	-0.02	0.21		-0.09 ~ 0.49	0.02 ± 0.01	0.05 ~ -0.01	0.02 ~ 0.02	-0.39	-0.48	0.000	***	-0.63 ~ -0.28
		55%	FP	69	0.00 ± 0.02													
			Est2	69	0.03 ± 0.02	0.87	-0.02	0.43		-0.10 ~ 0.74	0.02 ± 0.02	0.05 ~ -0.01	0.03 ~ 0.02	-0.31	-0.25	0.041	*	-0.46 ~ -0.01
		65%	FP	55	0.04 ± 0.03													
			Est2	55	0.06 ± 0.04	0.87	-0.01	0.78	††	0.00 ~ 0.93	0.02 ± 0.01	0.05 ~ -0.01	0.02 ~ 0.02	0.26	0.11	0.424	n.s.	-0.16 ~ 0.36
		75%	FP	29	0.08 ± 0.03													
			Est2	29	0.11 ± 0.04	0.79	0.00	0.71	†	-0.03 ~ 0.90	0.02 ± 0.02	0.06 ~ -0.01	0.03 ~ 0.02	0.34	0.18	0.343	n.s.	-0.20 ~ 0.51
	push-off	85%	FP	18	0.07 ± 0.03													
			Est2	18	0.10 ± 0.04	0.75	-0.01	0.64	†	-0.06 ~ 0.91	0.03 ± 0.01	0.06 ~ 0.01	0.04 ~ 0.03	1.86	0.69	0.002	**	0.31 ~ 0.87
		95%	FP	16	0.02 ± 0.01													
			Est2	16	0.04 ± 0.02	0.50	0.00	0.36		-0.11 ~ 0.74	0.02 ± 0.01	0.04 ~ -0.01	0.03 ~ 0.01	0.41	0.38	0.144	n.s.	-0.15 ~ 0.73
Z'	contact	5%	FP	36	0.43 ± 0.21													
			Est2	36	0.43 ± 0.21	1.00	0.00	1.00	†††	1.00 ~ 1.00	0.00 ± 0.00	0.01 ~ -0.01	0.00 ~ 0.00	-6.05	-0.09	0.604	n.s.	-0.41 ~ 0.25
		15%	FP	61	0.84 ± 0.16													
			Est2	61	0.84 ± 0.16	1.00	0.00	1.00	†††	1.00 ~ 1.00	0.00 ± 0.01	0.01 ~ -0.01	0.00 ~ 0.00	0.34	0.01	0.922	n.s.	-0.24 ~ 0.26
	single-support	25%	FP	80	0.96 ± 0.11													
			Est2	80	0.96 ± 0.11	1.00	0.00	1.00	†††	1.00 ~ 1.00	0.00 ± 0.00	0.01 ~ -0.01	0.00 ~ 0.00	0.51	0.02	0.864	n.s.	-0.20 ~ 0.24
		35%	FP	83	0.96 ± 0.08													
			Est2	83	0.96 ± 0.08	0.99	0.00	1.00	†††	0.97 ~ 1.00	0.01 ± 0.01	0.02 ~ -0.01	0.01 ~ 0.00	1.29	0.08	0.445	n.s.	-0.13 ~ 0.29
		45%	FP	77	1.00 ± 0.08													
			Est2	77	1.01 ± 0.08	0.97	0.03	0.99	†††	0.93 ~ 1.00	0.01 ± 0.01	0.02 ~ -0.01	0.01 ~ 0.01	4.12	0.35	0.002	**	0.13 ~ 0.53
		55%	FP	69	1.03 ± 0.10													
			Est2	69	1.04 ± 0.10	0.98	0.01	0.99	†††	0.91 ~ 1.00	0.01 ± 0.01	0.02 ~ -0.01	0.01 ~ 0.01	2.80	0.21	0.091	n.s.	-0.03 ~ 0.42
		65%	FP	55	1.03 ± 0.12													
			Est2	55	1.04 ± 0.12	0.99	0.00	1.00	†††	0.94 ~ 1.00	0.01 ± 0.01	0.02 ~ 0.00	0.01 ~ 0.01	3.13	0.17	0.214	n.s.	-0.10 ~ 0.42
		75%	FP	29	0.95 ± 0.12													
			Est2	29	0.95 ± 0.13	0.99	0.01	1.00	†††	0.99 ~ 1.00	0.00 ± 0.01	0.02 ~ -0.02	0.00 ~ 0.00	0.49	0.04	0.824	n.s.	-0.33 ~ 0.40
	push-off	85%	FP	18	0.52 ± 0.18													
			Est2	18	0.50 ± 0.18	0.99	0.02	0.99	†††	0.65 ~ 1.00	-0.02 ± 0.01	0.00 ~ -0.04	-0.01 ~ -0.03	1.02	0.07	0.797	n.s.	-0.42 ~ 0.52
		95%	FP	16	0.17 ± 0.06													
			Est2	16	0.16 ± 0.06	1.03	0.01	0.92	†††	0.02 ~ 0.98	-0.01 ± 0.01	0.00 ~ -0.03	-0.01 ~ -0.02	-2.74	-0.32	0.223	n.s.	-0.70 ~ 0.22

†††: ICC > 0.9, ††: ICC > 0.75, †: ICC ≧ 0.5, ***: p < 0.001, **: p < 0.01, *: p < 0.05, n.s.: no significant

Table S3

The sample size, mean ± standard deviation, regression slope and intercept between true and estimated values, ICC, bias, precision, and Bland-Altman analysis results for the X', Y', and Z' components of ground reaction force at each 10% interval time points for the left-leg during curve skating, calculated using FP and Est2. The magnitude of the force (N) was expressed as a value divided by the body weight (N).

	phase	% time		n (trial)	mean ± S.D. (no unit)	slope	intercept	ICC (2.1)		ICC (2.1) 95%CI	Bland-Altman analysis: bias ± precision	LoA	95%CI of bias	Bland-Altman plot: slope	r	*p*		95%CI
X'	contact	5%	FP	14	0.00 ± 0.05													
			Est2	14	0.02 ± 0.04	1.14	-0.03	0.78	††	-0.04 ~ 0.95	0.03 ± 0.02	0.06 ~ -0.01	0.04 ~ 0.02	-1.21	-0.50	0.072	n.s.	-0.81 ~ 0.07
		15%	FP	45	0.01 ± 0.06													
			Est2	45	0.05 ± 0.06	0.97	-0.04	0.76	††	-0.05 ~ 0.94	0.04 ± 0.02	0.08 ~ 0.01	0.05 ~ 0.04	-0.18	-0.05	0.738	n.s.	-0.34 ~ 0.25
	single-support	25%	FP	51	0.04 ± 0.07													
			Est2	51	0.09 ± 0.07	0.88	-0.04	0.73	†	-0.05 ~ 0.91	0.05 ± 0.03	0.11 ~ -0.01	0.06 ~ 0.04	0.09	0.04	0.778	n.s.	-0.24 ~ 0.31
		35%	FP	56	0.07 ± 0.07													
			Est2	56	0.11 ± 0.07	0.86	-0.03	0.70	†	-0.02 ~ 0.89	0.05 ± 0.03	0.11 ~ -0.02	0.05 ~ 0.04	0.03	0.02	0.909	n.s.	-0.25 ~ 0.28
		45%	FP	66	0.10 ± 0.06													
			Est2	66	0.15 ± 0.07	0.79	-0.02	0.63	†	-0.06 ~ 0.86	0.05 ± 0.04	0.12 ~ -0.02	0.06 ~ 0.04	0.15	0.09	0.479	n.s.	-0.16 ~ 0.32
		55%	FP	68	0.17 ± 0.08													
			Est2	68	0.23 ± 0.08	0.83	-0.02	0.73	†	-0.05 ~ 0.91	0.06 ± 0.04	0.13 ~ -0.02	0.07 ~ 0.05	0.38	0.18	0.142	n.s.	-0.06 ~ 0.40
		65%	FP	65	0.26 ± 0.09													
			Est2	65	0.33 ± 0.09	0.92	-0.05	0.68	†	-0.07 ~ 0.90	0.07 ± 0.03	0.14 ~ 0.01	0.08 ~ 0.07	0.01	0.00	0.976	n.s.	-0.24 ~ 0.25
		75%	FP	56	0.33 ± 0.09													
			Est2	56	0.44 ± 0.09	0.95	-0.09	0.53	†	-0.04 ~ 0.85	0.11 ± 0.03	0.18 ~ 0.05	0.12 ~ 0.10	-0.17	-0.06	0.638	n.s.	-0.32 ~ 0.20
	push-off	85%	FP	22	0.38 ± 0.10													
			Est2	22	0.49 ± 0.10	0.95	-0.08	0.66	†	-0.03 ~ 0.92	0.10 ± 0.03	0.15 ~ 0.05	0.11 ~ 0.09	0.25	0.06	0.782	n.s.	-0.37 ~ 0.47
		95%	FP	31	0.15 ± 0.08													
			Est2	31	0.19 ± 0.09	0.82	0.00	0.87	††	0.06 ~ 0.97	0.04 ± 0.02	0.08 ~ -0.01	0.05 ~ 0.03	1.94	0.58	0.001	***	0.27 ~ 0.77
Y'	contact	5%	FP	14	-0.01 ± 0.01													
			Est2	14	0.00 ± 0.00	-0.57	-0.01	-0.21		-0.71 ~ 0.37	0.00 ± 0.01	0.02 ~ -0.02	0.01 ~ 0.00	-0.28	-0.68	0.008	**	-0.88 ~ -0.20
		15%	FP	45	-0.02 ± 0.01													
			Est2	45	0.00 ± 0.00	0.85	-0.02	0.08		-0.06 ~ 0.27	0.02 ± 0.01	0.04 ~ 0.00	0.02 ~ 0.02	-0.47	-0.71	0.000	***	-0.83 ~ -0.53
	single-support	25%	FP	51	-0.03 ± 0.01													
			Est2	51	0.01 ± 0.01	-0.19	-0.02	-0.01		-0.06 ~ 0.06	0.03 ± 0.02	0.06 ~ 0.00	0.03 ~ 0.03	-0.29	-0.63	0.000	***	-0.77 ~ -0.42
		35%	FP	56	-0.02 ± 0.02													
			Est2	56	0.01 ± 0.01	0.84	-0.03	0.10		-0.07 ~ 0.31	0.03 ± 0.02	0.06 ~ 0.00	0.04 ~ 0.03	-0.45	-0.63	0.000	***	-0.76 ~ -0.43
		45%	FP	66	-0.01 ± 0.02													
			Est2	66	0.03 ± 0.01	0.62	-0.03	0.14		-0.06 ~ 0.42	0.04 ± 0.01	0.06 ~ 0.01	0.04 ~ 0.03	-0.13	-0.14	0.274	n.s.	-0.37 ~ 0.11
		55%	FP	68	0.00 ± 0.02													
			Est2	68	0.05 ± 0.03	0.70	-0.03	0.26		-0.06 ~ 0.62	0.05 ± 0.02	0.08 ~ 0.01	0.05 ~ 0.04	0.09	0.07	0.568	n.s.	-0.17 ~ 0.30
		65%	FP	65	0.03 ± 0.03													
			Est2	65	0.09 ± 0.03	0.75	-0.04	0.29		-0.03 ~ 0.67	0.06 ± 0.02	0.10 ~ 0.03	0.07 ~ 0.06	0.44	0.26	0.040	*	0.01 ~ 0.47
		75%	FP	56	0.05 ± 0.04													
			Est2	56	0.14 ± 0.04	0.74	-0.05	0.27		-0.03 ~ 0.64	0.08 ± 0.02	0.13 ~ 0.04	0.09 ~ 0.08	0.56	0.31	0.021	*	0.05 ~ 0.53
	push-off	85%	FP	22	0.07 ± 0.04													
			Est2	22	0.15 ± 0.05	0.74	-0.04	0.35		-0.03 ~ 0.75	0.08 ± 0.02	0.13 ~ 0.04	0.09 ~ 0.08	0.92	0.45	0.037	*	0.02 ~ 0.73
		95%	FP	31	0.03 ± 0.03													
			Est2	31	0.07 ± 0.04	0.59	-0.01	0.56	†	-0.09 ~ 0.85	0.04 ± 0.02	0.08 ~ 0.00	0.04 ~ 0.03	1.42	0.81	0.000	***	0.64 ~ 0.90
Z'	contact	5%	FP	14	0.34 ± 0.19													
			Est2	14	0.34 ± 0.19	1.00	0.00	1.00	†††	1.00 ~ 1.00	0.00 ± 0.00	0.01 ~ -0.01	0.00 ~ 0.00	-6.40	-0.10	0.731	n.s.	-0.60 ~ 0.46
		15%	FP	45	0.80 ± 0.20													
			Est2	45	0.80 ± 0.20	1.00	0.00	1.00	†††	1.00 ~ 1.00	0.00 ± 0.00	0.00 ~ -0.01	0.00 ~ 0.00	0.72	0.01	0.944	n.s.	-0.28 ~ 0.30
	single-support	25%	FP	51	1.00 ± 0.13													
			Est2	51	1.00 ± 0.13	1.00	0.00	1.00	†††	1.00 ~ 1.00	0.00 ± 0.00	0.01 ~ -0.01	0.00 ~ 0.00	-4.62	-0.15	0.290	n.s.	-0.41 ~ 0.13
		35%	FP	56	1.01 ± 0.08													
			Est2	56	1.01 ± 0.08	1.00	0.00	1.00	†††	0.99 ~ 1.00	0.00 ± 0.01	0.01 ~ -0.01	0.00 ~ -0.01	-1.16	-0.07	0.592	n.s.	-0.33 ~ 0.19
		45%	FP	66	1.04 ± 0.11													
			Est2	66	1.03 ± 0.11	0.99	0.02	1.00	†††	0.97 ~ 1.00	-0.01 ± 0.01	0.01 ~ -0.02	-0.01 ~ -0.01	1.43	0.10	0.409	n.s.	-0.14 ~ 0.34
		55%	FP	68	1.02 ± 0.11													
			Est2	68	1.01 ± 0.11	0.99	0.02	0.99	†††	0.87 ~ 1.00	-0.01 ± 0.01	0.01 ~ -0.04	-0.01 ~ -0.02	0.19	0.02	0.873	n.s.	-0.22 ~ 0.26
		65%	FP	65	1.01 ± 0.11													
			Est2	65	0.98 ± 0.11	0.98	0.05	0.96	†††	0.18 ~ 0.99	-0.03 ± 0.01	0.00 ~ -0.05	-0.02 ~ -0.03	0.82	0.11	0.401	n.s.	-0.14 ~ 0.34
		75%	FP	56	0.97 ± 0.12													
			Est2	56	0.91 ± 0.11	1.01	0.04	0.89	††	-0.02 ~ 0.98	-0.05 ± 0.02	-0.02 ~ -0.09	-0.05 ~ -0.06	-1.19	-0.17	0.214	n.s.	-0.41 ~ 0.10
	push-off	85%	FP	22	0.82 ± 0.15													
			Est2	22	0.75 ± 0.14	1.04	0.03	0.90	††	-0.01 ~ 0.98	-0.07 ± 0.02	-0.03 ~ -0.10	-0.06 ~ -0.08	-3.23	-0.40	0.064	n.s.	-0.70 ~ 0.03
		95%	FP	31	0.28 ± 0.12													
			Est2	31	0.24 ± 0.11	1.12	0.00	0.95	†††	0.22 ~ 0.99	-0.03 ± 0.02	0.01 ~ -0.07	-0.02 ~ -0.04	-4.20	-0.70	0.000	***	-0.84 ~ -0.46

†††: ICC > 0.9, ††: ICC > 0.75, †: ICC ≧ 0.5, ***: p < 0.001, **: p < 0.01, *: p < 0.05, n.s.: no significant

Table S4

The sample size, mean ± standard deviation, regression slope and intercept between true and estimated values, ICC, bias, precision, and Bland-Altman analysis results for the X', Y', and Z' components of ground reaction force at each 10% interval time points for the left-leg during curve skating, calculated using FP and Est2C. The magnitude of the force (N) was expressed as a value divided by the body weight (N).

	phase	% time		n	mean S.D.	slope	intercept	ICC (2.1)		Bland-Altman analysis						
				(trial)	(no unit)				95%CI	bias precision	LoA	95%CI of bias	Bland-Altman plot			
													slope	r	*p*	95%CI
X'	contact	5%	FP	12	0.00 ± 0.05											
			Est2C	12	-0.01 ± 0.03	1.36	0.01	0.88 ††	0.64 ~ 0.96	-0.01 ± 0.02	0.04 ~ -0.05	0.01 ~ -0.02	-1.42	-0.74	0.006 **	-0.92 ~ -0.25
		15%	FP	38	0.02 ± 0.06											
			Est2C	38	0.02 ± 0.05	1.14	-0.01	0.94 †††	0.88 ~ 0.97	0.01 ± 0.02	0.04 ~ -0.03	0.01 ~ 0.00	-1.53	-0.52	0.001 ***	-0.72 ~ -0.24
	single-support	25%	FP	44	0.05 ± 0.07											
			Est2C	44	0.05 ± 0.06	1.03	0.00	0.89 ††	0.81 ~ 0.94	0.00 ± 0.03	0.06 ~ -0.06	0.01 ~ -0.01	-0.62	-0.30	0.049 *	-0.54 ~ 0.00
		35%	FP	48	0.07 ± 0.07											
			Est2C	48	0.07 ± 0.06	0.97	0.01	0.85 ††	0.75 ~ 0.92	0.00 ± 0.03	0.06 ~ -0.07	0.01 ~ -0.01	-0.41	-0.23	0.124 n.s.	-0.48 ~ 0.07
		45%	FP	59	0.11 ± 0.06											
			Est2C	59	0.11 ± 0.06	0.91	0.01	0.82 ††	0.71 ~ 0.89	0.00 ± 0.04	0.07 ~ -0.07	0.01 ~ -0.01	-0.27	-0.17	0.199 n.s.	-0.41 ~ 0.09
		55%	FP	61	0.18 ± 0.08											
			Est2C	61	0.17 ± 0.07	0.95	0.02	0.89 ††	0.81 ~ 0.93	-0.01 ± 0.03	0.06 ~ -0.08	0.00 ~ -0.02	-0.29	-0.14	0.290 n.s.	-0.38 ~ 0.12
		65%	FP	59	0.27 ± 0.08											
			Est2C	59	0.26 ± 0.07	1.04	0.00	0.91 †††	0.84 ~ 0.95	-0.01 ± 0.03	0.05 ~ -0.07	0.00 ~ -0.02	-0.70	-0.29	0.025 *	-0.51 ~ -0.04
		75%	FP	51	0.34 ± 0.09											
			Est2C	51	0.35 ± 0.08	1.08	-0.04	0.92 †††	0.85 ~ 0.95	0.01 ± 0.03	0.07 ~ -0.05	0.02 ~ 0.00	-0.96	-0.37	0.007 **	-0.59 ~ -0.11
	push-off	85%	FP	21	0.39 ± 0.10											
			Est2C	21	0.39 ± 0.09	1.08	-0.03	0.97 †††	0.93 ~ 0.99	0.00 ± 0.02	0.04 ~ -0.05	0.01 ~ -0.01	-1.68	-0.45	0.049 *	-0.74 ~ 0.01
		95%	FP	30	0.15 ± 0.08											
			Est2C	30	0.13 ± 0.08	0.97	0.03	0.94 †††	0.45 ~ 0.98	-0.02 ± 0.02	0.02 ~ -0.06	-0.01 ~ -0.03	0.07	0.02	0.924 n.s.	-0.34 ~ 0.38
Y'	contact	5%	FP	12	0.00 ± 0.01											
			Est2C	12	-0.03 ± 0.00	-0.94	-0.03	-0.02	-0.09 ~ 0.17	-0.02 ± 0.01	0.00 ~ -0.04	-0.02 ~ -0.03	-0.40	-0.89	0.000 ***	-0.97 ~ -0.63
		15%	FP	38	-0.02 ± 0.01											
			Est2C	38	-0.03 ± 0.00	1.46	0.02	0.14	-0.10 ~ 0.40	-0.01 ± 0.01	0.01 ~ -0.03	0.00 ~ -0.01	-0.53	-0.89	0.000 ***	-0.94 ~ -0.79
	single-support	25%	FP	44	-0.02 ± 0.01											
			Est2C	44	-0.02 ± 0.00	-0.28	-0.03	-0.07	-0.36 ~ 0.24	0.00 ± 0.01	0.03 ~ -0.03	0.01 ~ 0.00	-0.41	-0.85	0.000 ***	-0.91 ~ -0.73
		35%	FP	48	-0.02 ± 0.02											
			Est2C	48	-0.02 ± 0.01	1.51	0.01	0.21	-0.09 ~ 0.47	0.00 ± 0.02	0.03 ~ -0.03	0.00 ~ 0.00	-0.56	-0.86	0.000 ***	-0.92 ~ -0.76
		45%	FP	59	-0.01 ± 0.02											
			Est2C	59	-0.01 ± 0.01	1.02	0.00	0.48	0.25 ~ 0.65	0.00 ± 0.01	0.03 ~ -0.03	0.00 ~ 0.00	-0.51	-0.63	0.000 ***	-0.76 ~ -0.44
		55%	FP	61	0.01 ± 0.02											
			Est2C	61	0.01 ± 0.02	1.14	0.00	0.65 †	0.47 ~ 0.77	0.00 ± 0.02	0.03 ~ -0.03	0.01 ~ 0.00	-0.61	-0.57	0.000 ***	-0.71 ~ -0.36
		65%	FP	59	0.03 ± 0.03											
			Est2C	59	0.03 ± 0.02	1.24	-0.01	0.80 ††	0.67 ~ 0.87	0.00 ± 0.02	0.03 ~ -0.03	0.00 ~ 0.00	-0.86	-0.59	0.000 ***	-0.74 ~ -0.39
		75%	FP	51	0.06 ± 0.04											
			Est2C	51	0.06 ± 0.03	1.18	-0.01	0.82 ††	0.70 ~ 0.89	0.00 ± 0.02	0.04 ~ -0.03	0.01 ~ 0.00	-0.85	-0.54	0.000 ***	-0.71 ~ -0.30
	push-off	85%	FP	21	0.07 ± 0.04											
			Est2C	21	0.07 ± 0.03	1.28	-0.02	0.88 ††	0.71 ~ 0.95	0.00 ± 0.02	0.04 ~ -0.04	0.01 ~ -0.01	-1.21	-0.61	0.004 **	-0.83 ~ -0.22
		95%	FP	30	0.03 ± 0.03											
			Est2C	30	0.02 ± 0.03	1.00	0.02	0.81 ††	-0.04 ~ 0.95	-0.02 ± 0.01	0.00 ~ -0.03	-0.01 ~ -0.02	-0.51	-0.17	0.358 n.s.	-0.50 ~ 0.20
Z'	contact	5%	FP	12	0.34 ± 0.20											
			Est2C	12	0.36 ± 0.20	1.01	-0.03	0.99 †††	0.14 ~ 1.00	0.03 ± 0.00	0.04 ~ 0.02	0.03 ~ 0.02	-31.76	-0.69	0.013 *	-0.90 ~ -0.16
		15%	FP	38	0.77 ± 0.20											
			Est2C	38	0.78 ± 0.20	1.02	-0.03	1.00 †††	0.30 ~ 1.00	0.02 ± 0.00	0.03 ~ 0.01	0.02 ~ 0.02	-32.27	-0.71	0.000 ***	-0.84 ~ -0.50
	single-support	25%	FP	44	0.98 ± 0.13											
			Est2C	44	0.99 ± 0.12	1.02	-0.04	0.99 †††	0.34 ~ 1.00	0.01 ± 0.01	0.02 ~ 0.00	0.02 ~ 0.01	-13.07	-0.53	0.000 ***	-0.71 ~ -0.27
		35%	FP	48	1.01 ± 0.09											
			Est2C	48	1.02 ± 0.09	1.02	-0.03	0.99 †††	0.34 ~ 1.00	0.01 ± 0.01	0.02 ~ 0.00	0.01 ~ 0.01	-4.35	-0.28	0.052 n.s.	-0.52 ~ 0.00
		45%	FP	59	1.04 ± 0.11											
			Est2C	59	1.05 ± 0.11	1.00	-0.01	0.99 †††	0.90 ~ 1.00	0.01 ± 0.01	0.03 ~ -0.01	0.01 ~ 0.01	-1.18	-0.09	0.518 n.s.	-0.33 ~ 0.17
		55%	FP	61	1.03 ± 0.11											
			Est2C	61	1.04 ± 0.11	1.00	-0.01	0.99 †††	0.99 ~ 1.00	0.00 ± 0.01	0.03 ~ -0.02	0.01 ~ 0.00	-0.90	-0.10	0.448 n.s.	-0.34 ~ 0.16
		65%	FP	59	1.02 ± 0.10											
			Est2C	59	1.01 ± 0.10	0.99	0.02	0.99 †††	0.96 ~ 0.99	-0.01 ± 0.01	0.02 ~ -0.04	-0.01 ~ -0.01	0.02	0.00	0.980 n.s.	-0.25 ~ 0.26
		75%	FP	51	0.98 ± 0.12											
			Est2C	51	0.94 ± 0.11	1.03	0.01	0.94 †††	0.06 ~ 0.99	-0.04 ± 0.02	0.00 ~ -0.07	-0.03 ~ -0.04	-1.85	-0.27	0.052 n.s.	-0.51 ~ 0.00
	push-off	85%	FP	21	0.82 ± 0.15											
			Est2C	21	0.78 ± 0.14	1.06	0.00	0.95 †††	0.05 ~ 0.99	-0.05 ± 0.02	-0.01 ~ -0.09	-0.04 ~ -0.06	-3.73	-0.48	0.031 *	-0.76 ~ -0.04
		95%	FP	30	0.28 ± 0.12											
			Est2C	30	0.27 ± 0.11	1.13	-0.03	0.98 †††	0.96 ~ 0.99	0.00 ± 0.02	0.04 ~ -0.05	0.00 ~ -0.01	-4.20	-0.74	0.000 ***	-0.87 ~ -0.51

†††: ICC > 0.9, ††: ICC > 0.75, †: ICC ≧ 0.5, ***: p < 0.001, **: p < 0.01, *: p < 0.05, n.s.: no significant
